# Prevalence of and Risk Factors Associated With Mental Health Symptoms Among the General Population in China During the Coronavirus Disease 2019 Pandemic

**DOI:** 10.1001/jamanetworkopen.2020.14053

**Published:** 2020-07-01

**Authors:** Le Shi, Zheng-An Lu, Jian-Yu Que, Xiao-Lin Huang, Lin Liu, Mao-Sheng Ran, Yi-Miao Gong, Kai Yuan, Wei Yan, Yan-Kun Sun, Jie Shi, Yan-Ping Bao, Lin Lu

**Affiliations:** 1 Institute of Mental Health, National Clinical Research Center for Mental Disorders, Key Laboratory of Mental Health and Peking University Sixth Hospital, Peking University, Beijing, China; 2 Savaid Medical School, University of Chinese Academy of Sciences, Beijing, China; 3 National Institute on Drug Dependence and Beijing Key Laboratory of Drug Dependence, Peking University, Beijing, China; 4 Peking University School of Public Health, Peking University, Beijing, China; 5 Department of Social Work and Social Administration, University of Hong Kong, Hong Kong, China; 6 Peking-Tsinghua Center for Life Sciences and PKU-IDG, McGovern Institute for Brain Research, Beijing, China

## Abstract

**Importance:**

People exposed to coronavirus disease 2019 (COVID-19) and a series of imperative containment measures could be psychologically stressed, yet the burden of and factors associated with mental health symptoms remain unclear.

**Objective:**

To investigate the prevalence of and risk factors associated with mental health symptoms in the general population in China during the COVID-19 pandemic.

**Design, Setting, and Participants:**

This large-sample, cross-sectional, population-based, online survey study was conducted from February 28, 2020, to March 11, 2020. It involved all 34 province-level regions in China and included participants aged 18 years and older. Data analysis was performed from March to May 2020.

**Main Outcomes and Measures:**

The prevalence of symptoms of depression, anxiety, insomnia, and acute stress among the general population in China during the COVID-19 pandemic was evaluated using the Patient Health Questionnaire–9, Generalized Anxiety Disorder–7, Insomnia Severity Index, and Acute Stress Disorder Scale. Logistic regression analyses were used to explore demographic and COVID-19–related risk factors.

**Results:**

Of 71 227 individuals who clicked on the survey link, 56 932 submitted the questionnaires, for a participation rate of 79.9%. After excluding the invalid questionnaires, 56 679 participants (mean [SD] age, 35.97 [8.22] years; 27 149 men [47.9%]) were included in the study; 39 468 respondents (69.6%) were aged 18 to 39 years. During the COVID-19 pandemic, the rates of mental health symptoms among the survey respondents were 27.9% (95% CI, 27.5%-28.2%) for depression, 31.6% (95% CI, 31.2%-32.0%) for anxiety, 29.2% (95% CI, 28.8%-29.6%) for insomnia, and 24.4% (95% CI, 24.0%-24.7%) for acute stress. Participants with confirmed or suspected COVID-19 and their family members or friends had a high risk for symptoms of depression (adjusted odds ratios [ORs], 3.27 [95% CI, 1.84-5.80] for patients; 1.53 [95% CI, 1.26-1.85] for family or friends), anxiety (adjusted ORs, 2.48 [95% CI, 1.43-4.31] for patients; 1.53 [95% CI, 1.27-1.84] for family or friends), insomnia (adjusted ORs, 3.06 [95% CI, 1.73-5.43] for patients; 1.62 [95% CI, 1.35-1.96] for family or friends), and acute stress (adjusted ORs, 3.50 [95% CI, 2.02-6.07] for patients; 1.77 [95% CI, 1.46-2.15] for family or friends). Moreover, people with occupational exposure risks and residents in Hubei province had increased odds of symptoms of depression (adjusted ORs, 1.96 [95% CI, 1.77-2.17] for occupational exposure; 1.42 [95% CI, 1.19-1.68] for Hubei residence), anxiety (adjusted ORs, 1.93 [95% CI, 1.75-2.13] for occupational exposure; 1.54 [95% CI, 1.30-1.82] for Hubei residence), insomnia (adjusted ORs, 1.60 [95% CI, 1.45-1.77] for occupational exposure; 1.20 [95% CI, 1.01-1.42] for Hubei residence), and acute stress (adjusted ORs, 1.98 [95% CI, 1.79-2.20] for occupational exposure; 1.49 [95% CI, 1.25-1.79] for Hubei residence). Both centralized quarantine (adjusted ORs, 1.33 [95% CI, 1.10-1.61] for depression; 1.46 [95% CI, 1.22-1.75] for anxiety; 1.63 [95% CI, 1.36-1.95] for insomnia; 1.46 [95% CI, 1.21-1.77] for acute stress) and home quarantine (adjusted ORs, 1.30 [95% CI, 1.25-1.36] for depression; 1.28 [95% CI, 1.23-1.34] for anxiety; 1.24 [95% CI, 1.19-1.30] for insomnia; 1.29 [95% CI, 1.24-1.35] for acute stress) were associated with the 4 negative mental health outcomes. Being at work was associated with lower risks of depression (adjusted OR, 0.85 [95% CI, 0.79-0.91]), anxiety (adjusted OR, 0.92 [95% CI, 0.86-0.99]), and insomnia (adjusted OR, 0.87 [95% CI, 0.81-0.94]).

**Conclusions and Relevance:**

The results of this survey indicate that mental health symptoms may have been common during the COVID-19 outbreak among the general population in China, especially among infected individuals, people with suspected infection, and people who might have contact with patients with COVID-19. Some measures, such as quarantine and delays in returning to work, were also associated with mental health among the public. These findings identify populations at risk for mental health problems during the COVID-19 pandemic and may help in implementing mental health intervention policies in other countries and regions.

## Key Points

**Question:**

What are the patterns of and factors associated with mental health conditions among the general population during the coronavirus disease 2019 (COVID-19) outbreak in China?

**Findings:**

In this survey study with 56 679 participants across all 34 province-level regions in China, 27.9% of participants had symptoms of depression, 31.6% had symptoms of anxiety, 29.2% had symptoms of insomnia, and 24.4% had symptoms of acute stress during the outbreak. Factors independently associated with negative mental health outcomes included having confirmed or suspected COVID-19, having a relative with confirmed or suspected COVID-19, having occupational exposure risks, living in Hubei province, and experiencing quarantine and delays in returning to work.

**Meaning:**

The mental health burden associated with COVID-19 is considerable among the general population of China, suggesting that mental health interventions are in urgent demand during the COVID-19 pandemic, especially for some at-risk populations.

## Teaser

This survey study investigates the prevalence of and risk factors associated with mental health symptoms in the general population in China during the coronavirus disease 2019 (COVID-19) pandemic.

## Introduction

In December 2019, the coronavirus disease 2019 (COVID-19) outbreak occurred and aroused global attention.^1^ It has infected more than 6.1 million people and caused 376 000 deaths worldwide across 216 countries, areas, or territories according to June 3, 2020, data from the World Health Organization.^2^ In contrast to previous epidemics and pandemics, such as severe acute respiratory syndrome and Middle East respiratory syndrome, COVID-19 is more contagious and spreads faster.^3^

The World Health Organization officially announced that COVID-19 was a pandemic and called on the whole world to work together to confront this infection.^4^ Compulsory measures, such as containment, quarantine, community control, and business and school closures, have been implemented in China and other countries to impede further escalation of the pandemic.^5,6,7,8^ Facing this large-scale infectious public health event and these enormous disruptions to daily life, people are under unprecedented pressure and are experiencing severe psychological distress.^9^

Few studies have investigated COVID-19–related mental health symptoms in the general population. One pilot study^10^ in China reported that nearly one-half of the respondents considered the mental health impact of COVID-19 as moderate or severe, and one-third exhibited anxiety symptoms. To set priorities for public health policies and implement effective health care interventions, the prevalence of mental health symptoms and factors that are associated with them during the COVID-19 outbreak need to be determined urgently. Thus, we conducted a large-sample, cross-sectional online survey study to investigate the prevalence of symptoms of depression, anxiety, insomnia, and acute stress and potential risk factors in the general population in China.

## Methods

### Study Design

This cross-sectional online study was conducted from February 28, 2020, to March 11, 2020. The study was approved by the ethics committee of Peking University Sixth Hospital (Institute of Mental Health). Written informed consent was received online before the respondents began the questionnaire. This study follows the American Association for Public Opinion Research (AAPOR) reporting guideline.

A self-designed online survey was released via the health page in the Chinese website Joybuy, a large ecommerce and information service platform that provides online health products and services in China. The survey link was posted on the website. We used a convenience sampling method. The registered members clicked the link on the platform and responded to the survey voluntarily until the convenience sample covered all 34 province-level regions in China. This was an anonymous survey, and confidentiality of data was ensured.

### Study Population

The respondents were all registered members of Joybuy. A total of 71 227 individuals clicked on the survey link, and 57 006 individuals commenced the survey, among whom 74 individuals refused to provide informed consent and 56 932 participants provided informed consent and submitted the questionnaires. One hundred fifty-six questionnaires that did not provide valid age information were excluded from the analysis. Ninety-seven respondents who were younger than 18 years were also excluded because obtaining online informed consent from their parents was not realistic under the present conditions.

### Measurements and Covariates

The survey lasted approximately 15 minutes and had 4 parts. The first part gathered demographic information of the participants, including gender, age, living area (urban vs rural), level of education, marital status, monthly family income, geographic region, history of chronic diseases, history of psychiatric disorders, family history of psychiatric disorders, and occupation. The second part asked epidemic-related questions. The third part evaluated isolation conditions and social attitudes toward the COVID-19 pandemic. The information about these 3 parts is listed in eTable 1 in the Supplement.

The fourth part of the questionnaire consisted of 4 standardized scales, including the Chinese versions of Patient Health Questionnaire–9,^11^ Generalized Anxiety Disorder–7,^12^ Insomnia Severity Index,^13^ and Acute Stress Disorder Scale,^14^ which measured symptoms of depression, anxiety, insomnia, and acute stress, respectively. The total scores of these scales were interpreted as follows: Patient Health Questionnaire–9, normal (0-4), mild (5-9), moderate (10-14), and severe (15-27) depression; Generalized Anxiety Disorder–7, normal (0-4), mild (5-9), moderate (10-14), and severe (15-21) anxiety; Insomnia Severity Index, normal (0-7), subthreshold (8-14), moderate (15-21), and severe (22-28) insomnia; and Acute Stress Disorder Scale, acute stress symptoms (a dissociative cluster score ≥9 and cumulative re-experiencing, avoidance, and arousal cluster scores ≥28). In the present study, cutoff scores of 5 for the Patient Health Questionnaire–9, 5 for the Generalized Anxiety Disorder–7 scale, and 8 for the Insomnia Severity Index were adopted to detect symptoms of depression, anxiety, and insomnia.

In this study, frontline workers are considered to be individuals who directly participated in the control of COVID-19, covering a wide range of occupations (eg, medicine, research, public health, media, security work, police, community work, emergency material delivery services, charity, construction, management, and psychological interventions). Frontline workers were presumed to have direct contact with patients with COVID-19. They were also presumed to possess personal protective equipment when they were in contact with patients with COVID-19. People with occupational exposure risks are individuals who did not necessarily participate in COVID-19 control but were required to contact people at work and could subjectively conceive of threats of being infected, including sanitary workers, taxi drivers, and supermarket workers, among others. They were presumed to be poorly equipped with self-protective measures.

### Statistical Analysis

Descriptive statistics were used to present demographic data. The prevalence of symptoms of depression, anxiety, insomnia, and acute stress was calculated using the aforementioned cutoff scores and reported as the percentages of cases in different populations. The 95% CIs were produced by the exact binomial methods. χ^2^ tests were used to compare the prevalence of mild-to-severe mental health symptoms in different populations. To explore factors potentially associated with depression, anxiety, insomnia, and acute stress, unadjusted logistic regression and multiple logistic regression analyses were performed, and odds ratios (ORs) and 95% CIs are presented. Two-sided Wald tests were conducted to determine whether the ORs in the regression models were statistically significant. All of the variables that were statistically significant in the unadjusted regression analysis and those that might convey important information were entered into the multivariable model. Detailed information of variables in the logistic regression is listed in eTable 2 in the Supplement. The level of significance was set to *P* < .05. All of the statistical analyses were performed using SPSS statistical software version 22 (IBM Corp). Data analysis was performed from March to May 2020.

## Results

### Demographic Characteristics

Data from a total of 56 679 eligible participants were included in the final analysis, for a participation rate of 79.9% (56 932 of 71 227 participants). The participants included in the analysis represented all 34 province-level regions in China. Of the total sample, 27 149 participants (47.9%) were male, and the mean (SD) age was 35.97 (8.22) years; 39 468 participants (69.6%) were aged 18 to 39 years. Of the total number of respondents, 47 139 (83.2%) had a university degree or higher, and 43 763 (77.2%) were married. Most of the participants (52 839 participants [93.2%]) lived in urban areas, and 2352 (4.1%) were from Hubei province (ie, the province most severely affected by COVID-19 in China). This survey included data from 100 individuals (0.2%) with confirmed or suspected cases of COVID-19, 219 (0.4%) people who were in close contact with patients with COVID-19, and 9725 (17.2%) frontline workers. Of the total number of respondents, 608 (1.1%) had at least 1 family member or friend who was infected with COVID-19, 17 587 (31.0%) had at least 1 family member or friend who was a frontline worker, 16 454 (29.0%) had quarantine experience, 19 754 (34.9%) were not yet back to work, and 2904 (5.1%) had occupational exposure risks. Additional demographic and epidemic-related characteristics are presented in Table 1.

**Table 1.** Descriptive Statistics of Demographic Characteristics and Epidemic-Related Information for the Total Sample

Factors	Participants, No. (%)
Overall	56 679 (100.0)
Gender	
Male	27 149 (47.9)
Female	29 530 (52.1)
Age, y	
18-39	39 468 (69.6)
≥40	17 211 (30.4)
Living area	
Urban	52 839 (93.2)
Rural	3840 (6.8)
Level of education	
Less than college	9540 (16.8)
College degree or higher	47 139 (83.2)
Marital status	
Married	43 763 (77.2)
Unmarried	12 916 (22.8)
Monthly family income, ¥^a^	
0-4999	13 016 (23.0)
5000-11 999	26 492 (46.7)
≥12 000	17 171 (30.3)
Geographical region, China	
Eastern	23 172 (40.9)
Northern	10 227 (18.0)
Northwest	1348 (2.4)
Northeast	3921 (6.9)
Central	4803 (8.5)
Southern	10 028 (17.7)
Southwest	3156 (5.6)
Missing values	24 (0.0)
History of chronic diseases	
Yes	3274 (5.8)
Unknown	1581 (2.8)
No	51 824 (91.4)
History of psychiatric disorders	
Yes	161 (0.3)
Unknown	381 (0.7)
No	56 137 (99.0)
Family history of psychiatric disorders	
Yes	396 (0.7)
Unknown	608 (1.1)
No	55 675 (98.2)
Are you infected with COVID-19?	
Confirmed or suspected cases	100 (0.2)
Not infected	56 579 (99.8)
Are you a frontline worker?	
Yes	9725 (17.2)
No	46 954 (82.8)
Have any of your family members or friends been infected with COVID-19?	
Yes	608 (1.1)
No	56 071 (98.9)
Are any of your family members or friends frontline workers?	
Yes	17 587 (31.0)
No	39 092 (69.0)
Have you come in close contact with patients infected with COVID-19?^b^	
Yes	219 (0.4)
No	56 460 (99.6)
Are you in Hubei province now?	
Yes	2352 (4.1)
No	54 327 (95.9)
Have you been to Hubei province in the past 2 mos?	
Yes	2452 (4.3)
No	54 227 (95.7)
Have you ever experienced quarantine?	
Centralized	587 (1.0)
Home	15 867 (28.0)
None	40 225 (71.0)
Are you back to work now?	
Work at home	7427 (13.1)
Work not at home	29 498 (52.0)
Not back to work	19 754 (34.9)
Are you likely to be exposed to other people at work?	
Exposed to patients infected with COVID-19	2904 (5.1)
Exposed to patients with other diseases	1597 (2.8)
Exposed to general people	19 740 (34.8)
Not at work, work at home, or without exposure to people at work	28 734 (50.7)
Missing values	3704 (6.5)

Abbreviation: COVID-19, coronavirus disease 2019.

^a^ As of June 10, 2020, 1 ¥ = $0.14 US.

^b^ Close contact is defined as people who have direct contact with confirmed or suspected cases of COVID-19, including family members who live with patients, colleagues who work with patients, classmates who study with patients, passengers who take the same vehicle as patients, and so on.

### Prevalence of Symptoms of Depression, Anxiety, Insomnia, and Acute Stress

The prevalence of symptoms for the 4 mental health conditions among the total sample was 27.9% (95% CI, 27.5%-28.2%) for depression (15 802 participants total, including 9688 participants [17.1%] with mild depression and 6114 participants [10.8%] with moderate-to-severe depression), 31.6% (95% CI, 31.2%-32.0%) for anxiety (17 897 participants total, including 12 026 participants [21.2%] with mild anxiety and 5871 participants [10.4%] with moderate-to-severe anxiety), 29.2% (95% CI, 28.8%-29.6%) for insomnia (16 564 participants total, including 13 308 participants [23.5%] with subthreshold insomnia and 3256 participants [5.7%] with moderate-to-severe insomnia), and 24.4% (95% CI, 24.0%-24.7%) for acute stress (13 817 participants). The prevalence of symptoms of the 4 mental health was high among patients with COVID-19 (depression, 75.0%; anxiety, 71.0%; insomnia, 68.0%; acute stress, 71.0%), frontline workers (depression, 30.4%; anxiety, 34.0%; insomnia, 32.4%; acute stress, 27.3%), family members or friends of patients with COVID-19 (depression, 46.7%; anxiety, 49.3%; insomnia, 48.4%; acute stress, 42.4%), family members or friends of frontline workers (depression, 29.3%; anxiety, 33.1%; insomnia, 31.6%; acute stress, 25.3%), residents of Hubei province (depression, 40.8%; anxiety, 44.9%; insomnia, 38.3%; acute stress, 33.7%), participants who were in close contact with patients with COVID-19 (depression, 53.9%; anxiety, 52.1%; insomnia, 56.2%; acute stress, 42.9%), participants who had been to Hubei province during the past 2 months (depression, 37.5%; anxiety, 41.5%; insomnia, 36.5%; acute stress, 30.2%), participants who experienced both centralized quarantine (depression, 38.0%; anxiety, 42.6%; insomnia, 43.3%; acute stress, 35.8%) and home quarantine (depression, 32.7%; anxiety, 36.4%; insomnia, 32.9%; acute stress, 28.3%), participants who had not yet returned to work (depression, 30.4%; anxiety, 33.6%; insomnia, 30.5%; acute stress, 25.5%), and participants who had occupational exposure risks (depression, 40.2%; anxiety, 45.0%; insomnia, 37.1%; acute stress, 37.8%). Additional details on the prevalence of the mental health symptoms in different populations are presented in Table 2.

**Table 2.** Prevalence of Symptoms of Depression, Anxiety, Insomnia, and Acute Stress in the Population Stratified by Epidemic-Related Factors

Variables	Depression^a^				Anxiety^b^				Insomnia^c^				Acute stress^d^	
	Participants, No. (%)		Total, No. (%) [95% CI]	*P* value^e^	Participants, No. (%)		Total, No. (%) [95% CI]	*P* value^e^	Participants, No. (%)		Total, No. (%) [95% CI]	*P* value^e^	Total, No. (%) [95% CI]	*P* value^e^
	Mild	Moderate to severe			Mild	Moderate to severe			Subthreshold	Moderate to severe				
Overall	9688 (17.1)	6114 (10.8)	15 802 (27.9) [27.5-28.2]		12 026 (21.2)	5871 (10.4)	17 897 (31.6) [31.2-32.0]		13 308 (23.5)	3256 (5.7)	16 564 (29.2) [28.8-29.6]		13 817 (24.4) [24.0-24.7]	
Gender														
Male	4781 (17.6)	3384 (12.5)	8165 (30.1) [29.5-30.6]	<.001	5666 (20.9)	2979 (11.0)	8645 (31.8) [31.3-32.4]	.19	6731 (24.8)	1686 (6.2)	8417 (31.0) [30.5-31.6]	<.001	7189 (26.5) [26.0-27.0]	<.001
Female	4907 (16.6)	2730 (9.2)	7637 (25.9) [25.4-26.4]		6360 (21.5)	2892 (9.8)	9252 (31.3) [30.8-31.9]		6577 (22.3)	1570 (5.3)	8147 (27.6) [27.1-28.1]		6628 (22.4) [22.0-22.9]	
Age, y														
18-39	7099 (18.0)	4750 (12.0)	11 849 (30.0) [29.6-30.5]	<.001	8724 (22.1)	4399 (11.1)	13 123 (33.2) [32.8-33.7]	<.001	9485 (24.0)	2318 (5.9)	11 803 (29.9) [29.5-30.4]	<.001	10 147 (25.7) [25.3-26.1]	<.001
≥40	2589 (15.0)	1364 (7.9)	3953 (23.0) [22.3-23.6]		3302 (19.2)	1472 (8.6)	4774 (27.7) [27.1-28.4]		3823 (22.2)	938 (5.5)	4761 (27.7) [27.0-28.3]		3670 (21.3) [20.7-21.9]	
Living area														
Urban	9015 (17.1)	5623 (10.6)	14 638 (27.7) [27.3-28.1]	<.001	11 179 (21.2)	5378 (10.2)	16 557 (31.3) [30.9-31.7]	<.001	12 407 (23.5)	3025 (5.7)	15 432 (29.2) [28.8-29.6]	.72	12 832 (24.3) [23.9-24.7]	.06
Rural	673 (17.5)	491 (12.8)	1164 (30.3) [28.9-31.8]		847 (22.1)	493 (12.8)	1340 (34.9) [33.4-36.4]		901 (23.5)	231 (6.0)	1132 (29.5) [28.0-30.9]		985 (25.7) [24.3-27.0]	
Are you infected with COVID-19?														
Confirmed or suspected cases	31 (31.0)	44 (44.0)	75 (75.0) [66.4-83.6]	<.001	44 (44.0)	27 (27.0)	71 (71.0) [62.0-80.0]	<.001	42 (42.0)	26 (26.0)	68 (68.0) [58.7-77.3]	<.001	71 (71.0) [62.0-80.0]	<.001
Not infected	9657 (17.1)	6070 (10.7)	15 727 (27.8) [27.4-28.2]		11 982 (21.2)	5844 (10.3)	17 826 (31.5) [31.1-31.9]		13 266 (23.4)	3230 (5.7)	16 496 (29.2) [28.8-29.5]		13 746 (24.3) [23.9-24.6]	
Are you a frontline worker?														
Yes	1705 (17.5)	1249 (12.8)	2954 (30.4) [29.5-31.3]	<.001	2142 (22.0)	1161 (11.9)	3303 (34.0) [33.0-34.9]	<.001	2471 (25.4)	678 (7.0)	3149 (32.4) [31.5-33.3]	<.001	2659 (27.3) [26.5-28.2]	<.001
No	7983 (17.0)	4865 (10.4)	12 848 (27.4) [27.0-27.8]		9884 (21.1)	4710 (10.0)	14 594 (31.1) [30.7-31.5]		10 837 (23.1)	2578 (5.5)	13 415 (28.6) [28.2-29.0]		11 158 (23.8) [23.4-24.1]	
Have any of your family members or friends been infected with COVID-19?														
Yes	129 (21.2)	155 (25.5)	284 (46.7) [42.7-50.7]	<.001	169 (27.8)	131 (21.5)	300 (49.3) [45.4-53.3]	<.001	213 (35.0)	81 (13.3)	294 (48.4) [44.4-52.3]	<.001	258 (42.4) [38.5-46.4]	<.001
No	9559 (17.0)	5959 (10.6)	15 518 (27.7) [27.3-28.0]		11 857 (21.1)	5740 (10.2)	17 597 (31.4) [31.0-31.8]		13 095 (23.4)	3175 (5.7)	16 270 (29.0) [28.6-29.4]		13 559 (24.2) [23.8-24.5]	
Are any of your family members or friends frontline workers?														
Yes	3114 (17.7)	2033 (11.6)	5147 (29.3) [28.6-29.9]	<.001	3859 (21.9)	1959 (11.1)	5818 (33.1) [32.4-33.8]	<.001	4424 (25.2)	1134 (6.4)	5558 (31.6) [30.9-32.3]	<.001	4452 (25.3) [24.7-26.0]	<.001
No	6574 (16.8)	4081 (10.4)	10 655 (27.3) [26.8-27.7]		8167 (20.9)	3912 (10.0)	12 079 (30.9) [30.4-31.4]		8884 (22.7)	2122 (5.4)	11 006 (28.2) [27.7-28.6]		9365 (24.0) [23.5-24.4]	
Have you come in close contact with patients infected with COVID-19?														
Yes	51 (23.3)	67 (30.6)	118 (53.9) [47.2-60.5]	<.001	65 (29.7)	49 (22.4)	114 (52.1) [45.4-58.7]	<.001	77 (35.2)	46 (21.0)	123 (56.2) [49.5-62.8]	<.001	94 (42.9) [36.3-49.5]	<.001
No	9637 (17.1)	6047 (10.7)	15 684 (27.8) [27.4-28.1]		11 961 (21.2)	5822 (10.3)	17 783 (31.5) [31.1-31.9]		13 231 (23.4)	3210 (5.7)	16 441 (29.1) [28.7-29.5]		13 723 (24.3) [24.0-24.7]	
Are you in Hubei province now?														
Yes	466 (19.8)	493 (21.0)	959 (40.8) [38.8-42.8]	<.001	562 (23.9)	493 (21.0)	1055 (44.9) [42.8-46.9]	<.001	662 (28.1)	239 (10.2)	901 (38.3) [36.3-40.3]	<.001	793 (33.7) [31.8-35.6]	<.001
No	9222 (17.0)	5621 (10.3)	14 843 (27.3) [26.9-27.7]		11 464 (21.1)	5378 (9.9)	16 842 (31.0) [30.6-31.4]		12 646 (23.3)	3017 (5.6)	15 663 (28.8) [28.5-29.2]		13 024 (24.0) [23.6-24.3]	
Have you been to Hubei province in the past 2 mos?														
Yes	481 (19.6)	439 (17.9)	920 (37.5) [35.6-39.4]	<.001	577 (23.5)	441 (18.0)	1018 (41.5) [39.6-43.5]	<.001	672 (27.4)	223 (9.1)	895 (36.5) [34.6-38.4]	<.001	740 (30.2) [28.4-32.0]	<.001
No	9207 (17.0)	5675 (10.5)	14 882 (27.4) [27.1-27.8]		11 449 (21.1)	5430 (10.0)	16 879 (31.1) [30.7-31.5]		12 636 (23.3)	3033 (5.6)	15 669 (28.9) [28.5-29.3]		13 077 (24.1) [23.8-24.5]	
Have you ever experienced quarantine?														
Centralized	105 (17.9)	118 (20.1)	223 (38.0) [34.1-41.9]	<.001	135 (23.0)	115 (19.6)	250 (42.6) [38.6-46.6]	<.001	172 (29.3)	82 (14.0)	254 (43.3) [39.3-47.3]	<.001	210 (35.8) [31.9-39.7]	<.001
Home	3058 (19.3)	2125 (13.4)	5183 (32.7) [31.9-33.4]		3696 (23.3)	2073 (13.1)	5769 (36.4) [35.6-37.1]		4106 (25.9)	1110 (7.0)	5216 (32.9) [32.1-33.6]		4492 (28.3) [27.6-29.0]	
None	6525 (16.2)	3871 (9.6)	10 396 (25.8) [25.4-26.3]		8195 (20.4)	3683 (9.2)	11 878 (29.5) [29.1-30.0]		9030 (22.4)	2064 (5.1)	11 094 (27.6) [27.1-28.0]		9115 (22.7) [22.3-23.1]	
Are you back to work now?														
Work at home	1305 (17.6)	785 (10.6)	2090 (28.1) [27.1-29.2]	<.001	1579 (21.3)	747 (10.1)	2326 (31.3) [30.3-32.4]	<.001	1823 (24.5)	418 (5.6)	2241 (30.2) [29.1-31.2]	<.001	1888 (25.4) [24.4-26.4]	<.001
Work not at home	4833 (16.4)	2875 (9.7)	7708 (26.1) [25.6-26.6]		6136 (20.8)	2801 (9.5)	8937 (30.3) [29.8-30.8]		6766 (22.9)	1538 (5.2)	8304 (28.2) [27.6-28.7]		6893 (23.4) [22.9-23.9]	
No	3550 (18.0)	2454 (12.4)	6004 (30.4) [29.8-31.0]		4311 (21.8)	2323 (11.8)	6634 (33.6) [32.9-34.2]		4719 (23.9)	1300 (6.6)	6019 (30.5) [29.8-31.1]		5036 (25.5) [24.9-26.1]	
Are you likely to be exposed to other people at work?														
Exposed to patients infected with COVID-19	619 (21.3)	549 (18.9)	1168 (40.2) [38.4-42.0]	<.001	774 (26.7)	533 (18.4)	1307 (45.0) [43.2-46.8]	<.001	818 (28.2)	259 (8.9)	1077 (37.1) [35.3-38.8]	<.001	1098 (37.8) [36.0-39.6]	<.001
Exposed to patients with other diseases	297 (18.6)	270 (16.9)	567 (35.5) [33.2-37.9]		383 (24.0)	228 (14.3)	611 (38.3) [35.9-40.6]		443 (27.7)	116 (7.3)	559 (35.0) [32.7-37.3]		523 (32.7) [30.4-35.1]	
Exposed to general people	3110 (15.8)	1589 (8.0)	4699 (23.8) [23.2-24.4]		3956 (20.0)	1575 (8.0)	5531 (28.0) [27.4-28.6]		4410 (22.3)	909 (4.6)	5319 (26.9) [26.3-27.6]		4100 (20.8) [20.2-21.3]	
Not at work, work at home, or without exposure to people at work	4880 (17.0)	2878 (10.0)	7758 (27.0) [26.5-27.5]		5937 (20.7)	2813 (9.8)	8750 (30.5) [29.9-31.0]		6523 (22.7)	1607 (5.6)	8130 (28.3) [27.8-28.8]		6568 (22.9) [22.4-23.3]	

Abbreviation: COVID-19, coronavirus disease 2019.

^a^ Scores of 5 to 9 on the Patient Health Questionnaire–9 were defined as mild depression, and scores of 10 or higher were defined as moderate-to-severe depression.

^b^ Scores of 5 to 9 on the Generalized Anxiety Disorder–7 were defined as mild anxiety, and scores of 10 or higher were defined as moderate-to-severe anxiety.

^c^ Scores of 8 to 14 on the Insomnia Severity Index were defined as subthreshold insomnia, and scores of 15 or higher were defined as moderate-to-severe insomnia.

^d^ Acute stress symptoms were defined as having an Acute Stress Disorder Scale dissociative cluster score of 9 or higher and cumulative re-experiencing, avoidance, and arousal cluster scores of 28 or higher.

^e^ χ2 tests were used to compare the prevalence of mild-to-severe mental health symptoms in different populations.

### Factors Associated With Symptoms of Depression, Anxiety, Insomnia, and Acute Stress

The results of the unadjusted analysis of demographic and epidemic-related variables are presented in eTable 3 in the Supplement. In the multivariable analysis, being younger than 40 years, having lower income levels, and having a personal history of psychiatric disorders were still found to be associated with the symptoms of depression, anxiety, insomnia, and acute stress. Male participants and unmarried people displayed a remarkably higher risk for depression, insomnia, and acute stress symptoms.

Individuals with confirmed or suspected COVID-19 had at least twice the risk for the 4 mental health symptoms compared with those not infected with COVID-19 (adjusted ORs, 3.27 [95% CI, 1.84-5.80] for depression, 2.48 [95% CI, 1.43-4.31] for anxiety, 3.06 [95% CI, 1.73-5.43] for insomnia, and 3.50 [95% CI, 2.02-6.07] for symptoms of acute stress). Family members or friends of patients with COVID-19 were also susceptible to symptoms of depression (adjusted OR, 1.53; 95% CI, 1.26-1.85), anxiety (adjusted OR, 1.53; 95% CI, 1.27-1.84), insomnia (adjusted OR, 1.62; 95% CI, 1.35-1.96), and acute stress symptoms (adjusted OR, 1.77; 95% CI, 1.46-2.15). In addition, associations were identified between potential occupational exposure risks to patients with COVID-19 and the 4 mental health outcomes (adjusted ORs, 1.96 [95% CI, 1.77-2.17] for depression, 1.93 [95% CI, 1.75-2.13] for anxiety, 1.60 [95% CI, 1.45-1.77] for insomnia, and 1.98 [95% CI, 1.79-2.20] for acute stress symptoms). Respondents with centralized quarantine or home quarantine experience had higher risks than those without quarantine experience of having depression (adjusted ORs, 1.33 [95% CI, 1.10-1.61] vs 1.30 [95% CI, 1.25-1.36]), anxiety (adjusted ORs, 1.46 [95% CI, 1.22-1.75] vs 1.28 [95% CI, 1.23-1.34]), insomnia (adjusted ORs, 1.63 [95% CI, 1.36-1.95] vs 1.24 [95% CI, 1.19-1.30]), and acute stress symptoms (adjusted ORs, 1.46 [95% CI, 1.21-1.77] vs 1.29 [95% CI, 1.24-1.35]). Hubei residents also demonstrated a higher risk than participants from other provinces, with adjusted ORs of 1.42 (95% CI, 1.19-1.68) for depression, 1.54 (95% CI, 1.30-1.82) for anxiety, 1.20 (95% CI, 1.01-1.42) for insomnia, and 1.49 (95% CI, 1.25-1.79) for acute stress symptoms. Nonetheless, being at work was associated with a lower risk of having symptoms of depression (adjusted OR, 0.85; 95% CI, 0.79-0.91), anxiety (adjusted OR, 0.92; 95% CI, 0.86-0.99), and insomnia (adjusted OR, 0.87; 95% CI, 0.81-0.94). Frontline workers had an elevated risk for insomnia (adjusted OR, 1.06; 95% CI, 1.00-1.12) and acute stress symptoms (adjusted OR, 1.08; 95% CI, 1.01-1.14), and being a close contact of a patient with COVID-19 remained statistically significant in the multivariable model of insomnia (1.55; 95% CI, 1.11-2.15). The detailed results of multivariable analysis are shown in Table 3.

**Table 3.** Multivariable Regression Analysis of Risk Factors Associated With Symptoms of Depression, Anxiety, Insomnia, and Acute Stress

Variables	Depression^a^		Anxiety^b^		Insomnia^c^		Acute stress^d^	
	AOR (95% CI)	*P* value	AOR (95% CI)	*P* value	AOR (95% CI)	*P* value	AOR (95% CI)	*P* value
Gender								
Male	1.21 (1.16-1.26)	<.001	NA	NA	1.13 (1.08-1.17)	<.001	1.20 (1.15-1.26)	<.001
Female	1 [Reference]		NA		1 [Reference]		1 [Reference]	
Age, y								
18-39	1.35 (1.29-1.42)	<.001	1.25 (1.20-1.30)	<.001	1.07 (1.02-1.12)	.003	1.21 (1.16-1.27)	<.001
≥40	1 [Reference]		1 [Reference]		1 [Reference]		1 [Reference]	
Living area								
Urban	1.10 (1.02-1.19)	.02	1.02 (0.95-1.10)	.62	NA	NA	NA	NA
Rural	1 [Reference]		1 [Reference]		NA		NA	
Level of education								
Less than college	0.97 (0.92-1.03)	.34	1.11 (1.06-1.17)	<.001	NA	NA	NA	NA
College or higher	1 [Reference]		1 [Reference]		NA		NA	
Marital status								
Married	0.83 (0.79-0.87)	<.001	1.03 (0.99-1.08)	.15	0.76 (0.73-0.80)	<.001	0.93 (0.88-0.98)	.003
Unmarried	1 [Reference]		1 [Reference]		1 [Reference]		1 [Reference]	
Monthly family income, ¥^e^								
0-4999	1.36 (1.29-1.44)	<.001	1.33 (1.26-1.41)	<.001	1.10 (1.04-1.16)	<.001	1.26 (1.19-1.34)	<.001
5000-11 999	1.19 (1.13-1.24)	<.001	1.14 (1.09-1.20)	<.001	1.06 (1.02-1.11)	.01	1.14 (1.08-1.19)	<.001
≥12 000	1 [Reference]		1 [Reference]		1 [Reference]		1 [Reference]	
History of chronic diseases								
Yes	1.26 (1.16-1.38)	<.001	NA	NA	1.53 (1.41-1.66)	<.001	1.19 (1.09-1.30)	<.001
Unknown	1.61 (1.44-1.80)	<.001	NA	NA	1.86 (1.67-2.08)	<.001	1.41 (1.25-1.58)	<.001
No	1 [Reference]		NA	NA	1 [Reference]		1 [Reference]	
History of psychiatric disorders								
Yes	2.11 (1.49-2.99)	<.001	1.72 (1.22-2.43)	.002	1.70 (1.20-2.41)	.003	1.47 (1.02-2.11)	.04
Unknown	1.80 (1.38-2.33)	<.001	1.91 (1.48-2.46)	<.001	1.60 (1.24-2.08)	<.001	1.85 (1.42-2.42)	<.001
No	1 [Reference]		1 [Reference]		1 [Reference]		1 [Reference]	
Family history of psychiatric disorders								
Yes	1.71 (1.37-2.14)	<.001	1.42 (1.13-1.77)	.002	1.66 (1.33-2.07)	<.001	1.22 (0.96-1.55)	.11
Unknown	1.37 (1.11-1.68)	.003	1.31 (1.07-1.60)	.009	1.26 (1.03-1.54)	.03	1.17 (0.95-1.46)	.14
No	1 [Reference]		1 [Reference]		1 [Reference]		1 [Reference]	
Are you infected with COVID-19?								
Confirmed or suspected cases	3.27 (1.84-5.80)	<.001	2.48 (1.43-4.31)	.001	3.06 (1.73-5.43)	<.001	3.50 (2.02-6.07)	<.001
Not infected	1 [Reference]		1 [Reference]		1 [Reference]		1 [Reference]	
Are you a frontline worker?								
Yes	1.03 (0.97-1.09)	.33	1.04 (0.99-1.10)	.13	1.06 (1.00-1.12)	.04	1.08 (1.01-1.14)	.02
No	1 [Reference]		1 [Reference]		1 [Reference]		1 [Reference]	
Have any of your family members or friends been infected with COVID-19?								
Yes	1.53 (1.26-1.85)	<.001	1.53 (1.27-1.84)	<.001	1.62 (1.35-1.96)	<.001	1.77 (1.46-2.15)	<.001
No	1 [Reference]		1 [Reference]		1 [Reference]		1 [Reference]	
Are any of your family members or friends frontline workers?								
Yes	1.07 (1.02-1.12)	.003	1.06 (1.01-1.10)	.01	1.14 (1.09-1.19)	<.001	1.01 (0.96-1.05)	.84
No	1 [Reference]		1 [Reference]		1 [Reference]		1 [Reference]	
Have you come in close contact with patients infected with COVID-19 ?								
Yes	1.31 (0.94-1.83)	.11	1.03 (0.74-1.44)	.85	1.55 (1.11-2.15)	.01	0.83 (0.58-1.18)	.29
No	1 [Reference]		1 [Reference]		1 [Reference]		1 [Reference]	
Are you in Hubei province now?								
Yes	1.42 (1.19-1.68)	<.001	1.54 (1.30-1.82)	<.001	1.20 (1.01-1.42)	.04	1.49 (1.25-1.79)	<.001
No	1 [Reference]		1 [Reference]		1 [Reference]		1 [Reference]	
Have you been to Hubei province in the past 2 mos?								
Yes	0.95 (0.80-1.13)	.56	0.94 (0.80-1.10)	.44	0.96 (0.82-1.14)	.67	0.80 (0.67-0.96)	.02
No	1 [Reference]		1 [Reference]		1 [Reference]		1 [Reference]	
Have you ever experienced quarantine?								
Centralized	1.33 (1.10-1.61)	.003	1.46 (1.22-1.75)	<.001	1.63 (1.36-1.95)	<.001	1.46 (1.21-1.77)	<.001
Home	1.30 (1.25-1.36)	<.001	1.28 (1.23-1.34)	<.001	1.24 (1.19-1.30)	<.001	1.29 (1.24-1.35)	<.001
None	1 [Reference]		1 [Reference]		1 [Reference]		1 [Reference]	
Are you back to work now?								
Work at home	0.87 (0.81-0.93)	<.001	0.92 (0.86-0.98)	.01	0.98 (0.91-1.04)	.47	0.99 (0.92-1.06)	.74
Work not at home	0.85 (0.79-0.91)	<.001	0.92 (0.86-0.99)	.03	0.87 (0.81-0.94)	<.001	0.98 (0.91-1.06)	.67
Not back to work	1 [Reference]		1 [Reference]		1 [Reference]		1 [Reference]	
Are you likely to be exposed to other people at work?								
Exposed to patients infected with COVID-19	1.96 (1.77-2.17)	<.001	1.93 (1.75-2.13)	<.001	1.60 (1.45-1.77)	<.001	1.98 (1.79-2.20)	<.001
Exposed to patients with other diseases	1.65 (1.46-1.87)	<.001	1.50 (1.33-1.69)	<.001	1.47 (1.30-1.66)	<.001	1.62 (1.43-1.84)	<.001
Exposed to general people	0.97 (0.90-1.05)	.44	0.98 (0.92-1.05)	.59	1.06 (0.99-1.14)	.11	.91 (0.84-0.98)	.01
Not at work, work at home, or without exposure to people at work	1 [Reference]		1 [Reference]		1 [Reference]		1 [Reference]	

Abbreviations: AOR, adjusted odds ratio; COVID-19, coronavirus disease 2019; NA, not available (variables that were not analyzed because they were not statistically significant in the unadjusted regression model).

^a^ Depression was defined as Patient Health Questionnaire–9 score of 5 or higher.

^b^ Anxiety was defined as Generalized Anxiety Disorder–7 score of 5 or higher.

^c^ Insomnia was defined as Insomnia Severity Index score of 8 or higher.

^d^ Acute stress was defined as having an Acute Stress Disorder Scale dissociative cluster score of 9 or higher and cumulative re-experiencing, avoidance, and arousal cluster score of 28 or higher.

^e^ As of June 10, 2020, 1 ¥ = $0.14 US.

## Discussion

The present study investigated the prevalence of and factors associated with mental health symptoms among the general population in China during the COVID-19 pandemic based on a nationwide, large-sample survey. Approximately one-quarter (24.4%) to one-third (31.6%) of respondents exhibited symptoms of depression, anxiety, insomnia, and acute stress. We also identified several psychologically vulnerable populations, such as individuals with confirmed or suspected COVID-19 and their relatives, respondents with occupational exposure risks, those who experienced quarantine, those who lived in Hubei province, and those who had not yet returned to work. These findings provide a comprehensive profile of psychological status in the general population in China during the COVID-19 outbreak and may contribute to developing population-specific mental health management and intervention strategies.

The prevalence of mental health symptoms in the present study is consistent with the results of an epidemiological study^15^ that was conducted in early February 2020 among the general population in China, which indicated that nearly 35% of the respondents manifested psychological distress during the COVID-19 outbreak. Another preliminary online survey^10^ that was performed in late January 2020 found that nearly one-third of respondents experienced moderate-to-severe mental health outcomes. The prevalence of these mental health symptoms during this period of the pandemic was higher than before the outbreak, during which rates of moderate-to-severe depression, moderate-to-severe anxiety, and insomnia in China were approximately 6.0%, 5.3%, and 15.0%, respectively.^16,17,18,19^ The prevalence of psychiatric morbidities during the severe acute respiratory syndrome epidemic in Singapore was reported to be 22.9%,^20^ and one-third of people exhibited psychological distress during the equine influenza outbreak in Australia.^21^ The prevalence of symptoms of depression, anxiety, insomnia, and acute stress in Hubei province was notable and was in accordance with previous studies of such psychiatric problems during other disasters.^22,23^ These findings indicate that severe emotional distress occurs among the general population during public health events and underscore the importance of preventing and treating mental health problems during the COVID-19 outbreak.

The present study identified several population groups who were likely to develop mental health symptoms. Compared with uninfected people, those with confirmed or suspected COVID-19 were 2 to 3 times more likely to report mental health symptoms. The prevalence of symptoms of the 4 mental health conditions was high among patients with COVID-19 (depression, 75.0%; anxiety, 71.0%; insomnia, 68.0%; acute stress, 71.0%). This is partially consistent with findings among patients with severe acute respiratory syndrome.^24^ The higher risk of mental health symptoms among patients experiencing an epidemic may be attributable to their distressing circumstances, physical pain, and adverse effects of medications that are used to treat infections.^25^ Moreover, family members and friends of patients with COVID-19 were susceptible to mental health disturbances. We found that nearly 50% of family members or friends of patients with COVID-19 experienced mild-to-severe mental health symptoms. Loved ones experiencing negative outcomes during disasters has been shown to be a strong catalyst of mental health disorders.^26,27^ In addition, relatives of patients with COVID-19 may worry about becoming infected themselves, may be quarantined, and may feel stigmatized, all of which may exacerbate psychological distress.^28,29^

The present study also found that people with occupational exposure risks reported greater symptoms of depression, anxiety, insomnia, and acute stress. People who work in high-risk environments often report more fatigue, health worries, and fear.^30^ COVID-19 may be symptomless during the incubation period, and its clinical manifestations can be easily confused with those of normal influenza.^31,32^ Therefore, people may understandably feel a threat of becoming infected by being exposed to general patients, thereby affecting their psychological well-being. However, in the present study, frontline work was not significantly associated with depression or anxiety. A previous study^33^ suggested that people at moderate infection risk (eg, individuals who might come in contact with patients with suspected cases) had more adverse mental health outcomes than those at high risk (eg, individuals who worked in infectious wards). The high infection risk group may be more aware of the risk, have better coping skills, have less uncertainty, and have more access to personal protective equipment and social support.

Another prominent finding was the substantial impact of quarantine experience on mental health, which is consistent with prior studies.^28^ Quarantine can contribute to poor mental health in both children and adults.^23,34^ People may experience fear of infection, frustration, and boredom during quarantine. Insufficient basic supplies and disruptions of information flow can increase both fear and anxiety.^28^ In the present study, both centralized quarantine and home quarantine enhanced the odds of adverse mental health outcomes. Moreover, centralized quarantine can have a more pernicious outcome because of fear of infection, being in an enclosed space, and being in an unfamiliar and crowded environment. The environment plays a vital role in maintaining healthy emotions and sleep.^35,36^ An unfamiliar and crowded environment may be a catalyst for the unique association between centralized quarantine and poor mental health status.

A high probability of symptoms of depression and anxiety was found among people who had not yet returned to work. Among people who had returned to work, those who worked at home had a higher risk of mental health symptoms compared with those who did not work at home. Being occupied with work activities can serve as a distraction from epidemic-related information. Interpersonal interactions that occur with conventional styles of work, in contrast to working at home, can ameliorate depression and lower the risk of mental disorders.^37,38^ Our results imply that accelerating people’s return to normal work may have a positive influence on mental health.

Some demographic factors may also influence mental health during the COVID-19 pandemic. Unmarried status and lower income were identified as risk factors for poor mental health outcomes, which is consistent with previous studies.^39,40,41^ However, opposite trends were observed for gender and age. The higher susceptibility of male participants to mental health symptoms during the pandemic may be attributable to their more frequent risky behaviors during epidemics (eg, going to crowded places or not wearing masks) and higher infective rate.^42,43^ The higher risk of mental health symptoms among younger people was also identified in this study, which is consistent with a previous study^44^ during the severe acute respiratory syndrome outbreak. This may be attributable to the fact that young adults frequently engage in social media and may be more exposed to misinformation online, which can trigger psychological distress.^45,46^ In addition, the proportion of self-reported history of mental disorders in this study was low, which might be attributed to stigma toward mental illnesses^47^ and the low recognition rate of mental health symptoms^48^ in China. We cannot exclude the possibility that the representativeness of the sample may influence the proportion of self-reported mental disorders.

### Strengths and Limitations

The strengths of this study include its extensive geographic coverage across China, large sample size, and the special study period. Participants with different demographic and COVID-19 epidemic–related characteristics were recruited from all 34 province-level regions in China. In addition, to the best of our knowledge, this is the first study that has systematically investigated the prevalence of and factors associated with mental health symptoms (ie, symptoms of depression, anxiety, insomnia, and acute stress) by standardized rating scales among the general population during the COVID-19 pandemic in China. Our findings may provide more helpful information for policy making, recognition of high-risk populations, and framework design for population-specific psychological crisis management.

Our study has several limitations. First, this was an online survey, and we used a convenience sampling method. Aside from posting our questionnaires on the main Joybuy website, we also sent proportional questionnaire links to registered members to increase the response rate. Although this study had extensive geographic coverage across China and a large sample size, it was conducted among internet users who were young and highly educated; thus, the representativeness of the sample might be limited. Second, the status of mental health symptoms was based on the respondents’ self-reports rather than clinical diagnoses. Third, this was a cross-sectional study. Therefore, associations between mental health symptoms and risk factors cannot necessarily be considered causal relationships. Fourth, the results may only reflect mental health status during the epidemic. Follow-up studies are needed to determine the possible long-term mental health outcomes associated with the COVID-19 pandemic.

## Conclusions

The prevalence of symptoms of depression, anxiety, insomnia, and acute stress was high in this sample of study participants from China during the COVID-19 pandemic, especially among patients with confirmed or suspected COVID-19 and their family members and friends, people with occupational exposure risks, and residents of Hubei province. Pervasive intervention measures, including quarantine and delays in returning to work, were closely associated with negative mental health outcomes. These findings suggest that the COVID-19 pandemic may have severe mental health repercussions. Population-specific mental health interventions are urgently needed to meet demand during this outbreak. Future studies are needed to explore the association of the COVID-19 pandemic with mental health in other countries and its long-term outcomes.

## Notes

Supplement. **eTable 1.** Summary of First Three Parts of the Questionnaire Related to This Study **eTable 2.** Factors Associated With Mental Health Symptoms Included in the Regression Model **eTable 3.** Unadjusted Regression Analysis of Risk Factors for Symptoms of Depression, Anxiety, Insomnia, and Acute Stress
