# Supplementary material for: Prevalence of and Risk Factors Associated With Mental Health Symptoms Among the General Population in China During the Coronavirus Disease 2019 Pandemic
Source: JAMA Netw Open. 2020 Jul 1;3(7):e2014053. doi: 10.1001/jamanetworkopen.2020.14053 (PMC7330717; doi:10.1001/jamanetworkopen.2020.14053)
Supplement: Supplement. — eTable 1. Summary of First Three Parts of the Questionnaire Related to This Study eTable 2. Factors Associated With Mental Health Symptoms Included in the Regression Model eTable 3. Unadjusted Regression Analysis of Risk Factors for Symptoms of Depression, Anxiety, Insomnia, and Acute Stress [file jamanetwopen-3-e2014053-s001.pdf]

Supplementary Online Content

Shi L, Lu Z-A, Que J-Y, et al. Prevalence of and risk factors associated with mental health symptoms among the general population in China during the coronavirus disease 2019 pandemic. *JAMA Netw Open*. 2020;3(7):e2014053.
doi:10.1001/jamanetworkopen.2020.14053

eTable 1. Summary of First Three Parts of the Questionnaire Related to This Study
eTable 2. Factors Associated With Mental Health Symptoms Included in the Regression Model
eTable 3. Unadjusted Regression Analysis of Risk Factors for Symptoms of Depression, Anxiety, Insomnia, and Acute Stress

This supplementary material has been provided by the authors to give readers additional information about their work.

© 2020 Shi L et al. *JAMA Network Open.*

**eTable 1. Summary of First Three Parts of the Questionnaire Related to This Study**		
**Questions**	**Types**	**Choices**
**Part One: Demographic information**		
Please select your gender:	Single choice	Male/Female
Please fill in your age (years):	Blank	/
Please fill in your current living geographical region:	Blank	/
Your current living area is:	Single choice	Urban/Rural
Please fill in your permanent geographical region:	Blank	/
Please select your highest level of education:	Single choice	Elementary school or below/Junior high school/Senior high school or vocational school/Bachelor degree or college diploma/Master degree or above
Please select your current marital status:	Single choice	Married/Unmarried
Please select your monthly family income (yuan):	Single choice	<1000/1000-2999/3000-4999/5000-7999/8000-11999/≥12000
Do you have a history of chronic diseases (hypertension, diabetes, cerebrovascular disease, heart diseases, and malignant tumors)?	Single choice	Yes/Unknown/No
Do you have a history of psychiatric disorders?	Single choice	Yes/Unknown/No
Please select the type of psychiatric disorder you suffer from: (this depends on "Yes" being checked on the last question)	Multiple choice	Depression/Bipolar disorder/ Schizophrenia/Anxiety/Obsessive-compulsive disorder/Attention deficit hyperactivity disorder/Others/Unknown
Do you have a family history of psychiatric disorders?	Single choice	Yes/Unknown/No
Have you ever had any sleep problems?	Single choice	Yes/No
Do you have the habit of smoking?	Single choice	Yes/No
Do you have the habit of drinking alcohol?	Single choice	Yes/No
Please select your current occupation:	Single choice	Ordinary full-time students/Medical students/ Graduates/Medical staff/Other medical workers/Scientific researchers/The police, the security guards/Workers in the transportation industry/Community workers or social workers/Other administrative staff/Clerical staff/Other agricultural personnel/Other commercial or service staff/Other professionals (e.g. accountants, lawyers, architects, journalists, etc.)/Other workers/Military personnel/Self-employment or freelancers/Unemployed/Others
**Part Two: Epidemic-related questions**		
Are you infected with COVID-19?	Single choice	Confirmed cases/Suspected cases/Not infected
Have any of your family members or friends been infected with COVID-19?	Single choice	Yes/No
Have you come in close contact with patients infected with COVID-19?	Single choice	Yes/No
Have you been to Hubei province in the past 2 mos?	Single choice	Yes/No

© 2020 Shi L et al. *JAMA Network Open.*

Please comment on your level of concern about the COVID-19 epidemic:	Enter number from 0 (very unconcerned) to 10 (very concerned)	/
Please select the main channels for you to obtain information about the COVID-19 epidemic?	Multiple choice	TV news/Network news/Search engines (e.g. Google, etc.)/Social platform (e.g. WeChat, chat group, ins, etc.)/Broadcast/Paper media/Newspaper brochure/Others
Please comment on your level of understanding of the COVID-19 epidemic:	Enter number from 0 (understand very little) to 10 (understand very well)	/
Are you a frontline worker of COVID-19?	Single choice	Yes/No
Are any of your family members or friends frontline workers?	Single choice	Yes/No
Are you back to work now?	Single choice	Yes/No
If you are currently working, what is your workplace? (this depends on "Yes" being checked in the last question)	Single choice	Work at home/Work not at home
Are you likely to be exposed to other people at work?	Single choice	Close contact (≤1.5 m) to patients infected with COVID-19/Non-close contact (＞1.5 m) with patients infected with COVID-19/Close contact (≤1.5 m) with patients with other diseases/Non-close contact (＞1.5 m) with patients with other diseases/Close contact (≤1.5 m) with general people/Non-close contact (＞1.5 m) with general people/Not at work, work at home, or without exposure to people at work
What is the impact of the COVID-19 pandemic on your work?	Multiple choice	Unemployment/Postponement of work/Impact on income/ Busier than ever/No impact/Others
**Part Three: Isolation conditions and social attitudes toward the COVID-19 pandemic**		
Was there any traffic control in your area during the pandemic?	Single choice	Yes/No
Do you live in a community that restricts people's access?	Single choice	Yes/No
Have you ever experienced quarantine?	Single choice	Centralized quarantine/Home quarantine/No quarantine
How long have you been quarantining? (this depends on "centralized quarantine" or "home quarantine" being checked on the last question)	Single choice	Fewer than 7 days/7-13 days/14 days/More than 14 days
Do you quarantine as required? (this depends on "centralized quarantine" or "home quarantine" being checked on the question "Have you ever experienced quarantine?")	Single choice	Yes/No
If you don't quarantine as required, what's the reason? (this depends on "No" being checked on the last question)	Multiple choice	I must go out under special circumstances/I don't need quarantine since I'm not infected /I cannot stand the feeling of boringness and loneliness due to quarantine/Others
To what extent are you worried about being infected with COVID-19?	Single choice	Not at all/Slight/Moderate/Significant/Severe

© 2020 Shi L et al. *JAMA Network Open.*

How long do you think the pandemic will take to be controlled?	Single choice	Fewer than 1 month/2-3 months/4-6 months/More than 6 months
What do you think are the difficulties in the current pandemic prevention and control?	Multiple choice	Shortage of protection materials/Insufficient medical personnel and medical resources/Patients/People under quarantine don't understand or cooperate/People are not fully aware of the importance of self-protection/Mental problems/Others
Are you stressed due to the local pandemic and various restriction policies?	Single choice	Yes/No
What's your main stressor?(this depends on "Yes" being checked on the last question)	Multiple choice	The severity of the pandemic/Various access restriction policies/Worries about their own and family members' safety/Worries about the safety of medical personnel working in frontline/Others
To what extent you feel stressed before the pandemic?	Enter the number from 0 (no stress) to 10 (extreme stress)	/
Would you like to learn psychological knowledge disseminated by media before the pandemic?	Single choice	Yes/No
Would you like to learn psychological intervention and therapy knowledge before the pandemic?	Single choice	Yes/No
To what extent the difficulty you encountered in finding information about psychological therapy and intervention before pandemic?	Enter the number from 0 (not difficult at all) to 10 (extremely difficult)	/
To what extent you feel stressed after the pandemic?	Enter the number from 0 (no stress) to 10 (extreme stress)	/
Would you like to learn psychological knowledge disseminated by media after the pandemic?	Single choice	Yes/No
Would you like to learn psychological intervention and therapy knowledge after the pandemic?	Single choice	Yes/No
To what extent the difficulty you encountered in finding information about psychological therapy and intervention after pandemic?	Enter the number from 0 (not difficult at all) to 10 (extremely difficult)	/
I would report and seek treatment if I was infected	Single choice	Yes/No
For what reason you would not report and seek treatment? (this depends on "No" being checked on the last question)	Multiple choice	Worries about the judgement of people/Worries about being labeled/Impact on later life/Invasion of privacy/Family factors (e.g. worries about parents or children, etc.)/Worries about treatment effects/Others
Are you in Hubei now?	Single choice	Yes, I'm in Wuhan/Yes, I'm in another city of Hubei/No

© 2020 Shi L et al. *JAMA Network Open.*

Did you return from Hubei to your current location?(this depends on "No" being checked on the last question)	Single choice	Yes, I returned from Wuhan /Yes, I returned from another city of Hubei/No
Social attitudes towards and perceived discrimination among people in the most severely affected area by COVID-19	Items and scales	/

eTable 2. Factors Associated With Mental Health Symptoms Included in the Regression Model.

Factors	Definitions	Types of variables	Reference category	Summarizing forms
**Gender**	In the following 2 categories: 1.male; 2.female	Categorical	Female	Odds ratio with a 95% confidence interval
**Age (years)**	In the following 2 categories: 1.18-39; 2.≥ 40	Categorical	≥ 40	Odds ratio with a 95% confidence interval
**Living area**	In the following 2 categories: 1.urban; 2.rural	Categorical	Rural	Odds ratio with a 95% confidence interval
**Level of education**	In the following 2 categories: 1. less than college; 2. college or higher	Categorical	College or higher	Odds ratio with a 95% confidence interval
**Marital status**	In the following 2 categories: 1.married; 2.unmarried	Categorical	Unmarried	Odds ratio with a 95% confidence interval
**Monthly family income(yuan)**	In the following 3 categories: 1.0-4999; 2.5000-11999; 3. ≥ 12000	Categorical	≥ 12000	Odds ratio with a 95% confidence interval
**History of chronic diseases**	Yes if the participant had a history of chronic diseases (including hypertension, diabetes, cardiovascular diseases, heart diseases and malignant tumors); unknown if participants reported to be unclear of their own history of chronic diseases; no otherwise	Categorical	Not with a history of chronic diseases	Odds ratio with a 95% confidence interval
**History of psychiatric disorders**	Yes if the participant had a history of psychiatric disorders; unknown if participants reported to be unclear of their own history of psychiatric disorders; no otherwise	Categorical	Not with a history of psychiatric disorders	Odds ratio with a 95% confidence interval
**Family history of psychiatric disorders**	Yes if the participant had family history of psychiatric disorders; unknown if participants reported to be unclear of their family history of psychiatric disorders; no otherwise	Categorical	Not with a family history of psychiatric disorders	Odds ratio with a 95% confidence interval

© 2020 Shi L et al. *JAMA Network Open.*

**Are you infected with COVID-19?**	In the following 2 categories: 1.confirmed or suspected cases of COVID-19; 2.not infected	Categorical	Not infected	Odds ratio with a 95% confidence interval
**Are you a frontline worker?**	Yes if the participant directly participated in the control of COVID-19, covering a wide range of occupations (e.g., medicine, research, public health, media, security work, police, community work, emergency material delivery services, charity, construction, management, and psychological interventions); no otherwise	Categorical	Not involved in frontline work of COVID-19	Odds ratio with a 95% confidence interval
**Have any of your family members or friends been infected with COVID-19?**	Yes if the participant reported to have at least a family member or friend infected with COVID-19 according to their known situation; no otherwise	Categorical	Not having any family member or friend infected with COVID-19	Odds ratio with a 95% confidence interval
**Are any of your family members or friends frontline workers?**	Yes if the participant reported to have at least a family member or friend directly participating in the control of COVID-19 according to their known situation; no otherwise	Categorical	Not having any family member or friend being frontline workers of COVID-19	Odds ratio with a 95% confidence interval
**Have you come in close contact with patients infected with COVID-19?**	Yes if the participant reported to have direct contact with confirmed or suspected cases of COVID-19 (including family members who lived with patients, colleagues who worked with patients, classmates who studied with patients, passengers who took the same vehicle as patients, etc.); no otherwise	Categorical	Not having come in close contact with patients infected with COVID-19	Odds ratio with a 95% confidence interval
**Are you in Hubei province now?**	Yes if the participant reported to be in Hubei province when completing the questionnaire; no otherwise	Categorical	Not in Hubei province at present	Odds ratio with a 95% confidence interval
**Have you been to Hubei province in the past 2 months?**	Yes if the participant reported to have been to Hubei province in the past 2 months; no otherwise	Categorical	Not been to Hubei province in the past 2 months	Odds ratio with a 95% confidence interval
**Have you ever experienced quarantine?**	In the following 3 categories: 1.centralized quarantine; 2.home quarantine; 3. no quarantine experience	Categorical	No quarantine experience	Odds ratio with a 95% confidence interval

© 2020 Shi L et al. *JAMA Network Open.*

**Are you back to work now?**	In the following 3 categories: 1.work at home; 2.work not at home; 3.not back to work	Categorical	Not back to work	Odds ratio with a 95% confidence interval
**Are you likely to be exposed to other people at work?**	In the following 4 categories: 1.exposed to patients infected with COVID-19 (eg. sanitory workers in the ward hospitalizing COVID-19 patients); 2. exposed to patients with other diseases (eg. ambulance drivers); 3. exposed to general people (eg. supermarket workers); 4. not at work, work at home, or without exposure to people at work	Categorical	Not at work, work at home, or without exposure to people at work	Odds ratio with a 95% confidence interval

h

eTable 3. Unadjusted Regression Analysis of Risk Factors for Symptoms of Depression, Anxiety, Insomnia, and Acute Stress

Variables	Depression[a]		Anxiety[b]		Insomnia[c]		Acute stress[d]	
	OR (95% CI)	*P* value	OR (95% CI)	*P* value	OR (95% CI)	*P* value	OR (95% CI)	*P* value
**Gender**								
Male	1.23 (1.19-1.28)	< .001	1.02 (0.99-1.06)	.19	1.18 (1.14-1.22)	< .001	1.25 (1.20-1.29)	< .001
Female	1 [Reference]		1 [Reference]		1 [Reference]		1 [Reference]	
**Age (years)**								
18-39	1.44 (1.38-1.50)	< .001	1.30 (1.25-1.35)	< .001	1.12 (1.07-1.16)	< .001	1.28 (1.22-1.33)	< .001
≥ 40	1 [Reference]		1 [Reference]		1 [Reference]		1 [Reference]	
**Living area**								
Urban	0.88 (0.82-0.95)	.001	0.85 (0.80-0.91)	< .001	0.99 (0.92-1.06)	.72	0.93 (0.86-1.00)	.06
Rural	1 [Reference]		1 [Reference]		1 [Reference]		1 [Reference]	
**Level of education**								
Less than ollege	1.06 (1.01-1.11)	.03	1.20 (1.15-1.26)	< .001	0.99 (0.94-1.03)	.55	1.03 (0.98-1.09)	.22
College or higher	1 [Reference]		1 [Reference]		1 [Reference]		1 [Reference]	
**Marital status**								
Married	0.71 (0.68-0.74)	< .001	0.92 (0.88-0.96)	< .001	0.72 (0.69-0.75)	< .001	0.82 (0.78-0.86)	< .001
Unmarried	1 [Reference]		1 [Reference]		1 [Reference]		1 [Reference]	
**Monthly family income (yuan)**								
0-4999	1.52 (1.44-1.60)	< .001	1.48 (1.41-1.55)	< .001	1.23 (1.17-1.29)	< .001	1.38 (1.31-1.46)	< .001
5000-11999	1.24 (1.19-1.30)	< .001	1.20 (1.15-1.25)	< .001	1.11 (1.06-1.16)	< .001	1.18 (1.13-1.24)	< .001
≥ 12000	1 [Reference]		1 [Reference]		1 [Reference]		1 [Reference]	
**History of chronic diseases**								
Yes	1.15 (1.07-1.24)	< .001	1.08 (1.00-1.16)	.06	1.51 (1.40-1.62)	< .001	1.13 (1.05-1.23)	.002
Unknown	1.87 (1.69-2.07)	< .001	1.68 (1.52-1.86)	< .001	2.14 (1.93-2.37)	< .001	1.63 (1.46-1.81)	< .001

© 2020 Shi L et al. *JAMA Network Open.*

No	1 [Reference]		1 [Reference]		1 [Reference]		1 [Reference]	
**History of psychiatric disorders**								
Yes	3.01 (2.21-4.10)	< .001	2.27 (1.67-3.10)	< .001	2.54 (1.87-3.47)	< .001	2.12 (1.55-2.91)	< .001
Unknown	3.51 (2.86-4.30)	<.001	2.69 (2.20-3.29)	<.001	3.07 (2.51-3.77)	< .001	2.87 (2.34-3.51)	< .001
No	1 [Reference]		1 [Reference]		1 [Reference]		1 [Reference]	
**Family history of psychiatric disorders**								
Yes	1.88 (1.54-2.29)	< .001	1.47 (1.20-1.80)	< .001	1.99 (1.63-2.42)	< .001	1.43 (1.16-1.77)	.001
Unknown	2.33 (1.99-2.74)	< .001	1.89 (1.61-2.22)	< .001	2.15 (1.84-2.53)	< .001	1.95 (1.65-2.30)	< .001
No	1 [Reference]		1 [Reference]		1 [Reference]		1 [Reference]	
**Are you infected with COVID-19?**								
Confirmed or suspected cases	7.79 (4.95-12.26)	< .001	5.32 (3.45-8.20)	< .001	5.16 (3.39-7.86)	< .001	7.63 (4.95-11.76)	< .001
Not infected	1 [Reference]		1 [Reference]		1 [Reference]		1 [Reference]	
**Are you a frontline worker?**								
Yes	1.16 (1.10-1.22)	< .001	1.14 (1.09-1.19)	< .001	1.20 (1.14-1.26)	< .001	1.21 (1.15-1.27)	< .001
No	1 [Reference]		1 [Reference]		1 [Reference]		1 [Reference]	
**Have any of your family members or friends been infected with COVID-19?**								
Yes	2.29 (1.95-2.69)	< .001	2.13 (1.82-2.50)	< .001	2.29 (1.95-2.69)	< .001	2.31 (1.97-2.72)	< .001
No	1 [Reference]		1 [Reference]		1 [Reference]		1 [Reference]	
**Are any of your family members or friends frontline workers?**								
Yes	1.10 (1.06-1.15)	< .001	1.11 (1.06-1.15)	< .001	1.18 (1.13-1.23)	< .001	1.08 (1.03-1.12)	< .001
No	1 [Reference]		1 [Reference]		1 [Reference]		1 [Reference]	
**Have you come in close contact with patients infected with COVID-19?**								
Yes	3.04 (2.33-3.96)	< .001	2.36 (1.81-3.08)	< .001	3.12 (2.39-4.08)	< .001	2.34 (1.79-3.06)	< .001
No	1 [Reference]		1 [Reference]		1 [Reference]		1 [Reference]	
**Are you in Hubei province now?**								
Yes	1.83 (1.68-1.99)	< .001	1.81 (1.67-1.97)	< .001	1.53 (1.41-1.67)	< .001	1.61 (1.48-1.76)	< .001

© 2020 Shi L et al. *JAMA Network Open.*

No	1 [Reference]		1 [Reference]		1 [Reference]		1 [Reference]	
**Have you been to Hubei province in the past 2 mos?**								
Yes	1.59 (1.46-1.73)	< .001	1.57 (1.45-1.71)	< .001	1.42 (1.30-1.54)	< .001	1.36 (1.25-1.49)	< .001
No	1 [Reference]		1 [Reference]		1 [Reference]		1 [Reference]	
**Have you ever experienced quarantine?**								
Centralized	1.76 (1.49-2.08)	< .001	1.77 (1.50-2.09)	< .001	2.00 (1.70-2.36)	< .001	1.90 (1.60-2.25)	< .001
Home	1.39 (1.34-1.45)	< .001	1.36 (1.31-1.42)	< .001	1.29 (1.24-1.34)	< .001	1.35 (1.29-1.41)	< .001
None	1 [Reference]		1 [Reference]		1 [Reference]		1 [Reference]	
**Are you back to work now?**								
Work at home	0.90 (0.85-0.95)	< .001	0.90 (0.85-0.96)	< .001	0.99 (0.93-1.05)	.64	1.00 (0.94-1.06)	.90
Work not at home	0.81(0.78-0.84)	< .001	0.86 (0.83-0.89)	< .001	0.89(0.86-0.93)	< .001	0.89(0.86-0.93)	< .001
Not back to work	1 [Reference]		1 [Reference]		1 [Reference]		1 [Reference]	
**Are you likely to be exposed to other people at work?**								
Exposed to patients infected with COVID-19	1.82 (1.68-1.97)	< .001	1.87 (1.73-2.02)	< .001	1.49 (1.38-1.62)	< .001	2.05 (1.89-2.22)	< .001
Exposed to patients with other diseases	1.49 (1.34-1.65)	< .001	1.42 (1.28-1.57)	< .001	1.37 (1.23-1.52)	< .001	1.64 (1.48-1.83)	< .001
Exposed to general people	0.85 (0.81-0.88)	< .001	0.89 (0.85-0.93)	< .001	0.94 (0.90-0.97)	.001	0.89 (0.85-0.93)	< .001
Not at work, work at home, or without exposure to people at work	1 [Reference]		1 [Reference]		1 [Reference]		1 [Reference]	

[a] Depression was defined as Patient Health Questionnaire–9 score ≥ 5. [b] Anxiety was defined as Generalized Anxiety Disorder–7 score ≥ 5. [c] Insomnia was defined as Insomnia Severity Index score ≥ 8. [d] Acute stress was defined as having an Acute Stress Disorder Scale dissociative cluster score ≥ 9 and cumulative re-experiencing, avoidance, and arousal cluster score ≥ 28.

© 2020 Shi L et al. *JAMA Network Open.*
